# Serum Uric Acid and Hyperuricemia Associate with Coronary Artery Disease among Postmenopausal Women

**DOI:** 10.31083/j.rcm2307222

**Published:** 2022-06-24

**Authors:** Qianyun Guo, Yang Liu, Xunxun Feng, Jiaqi Yang, Guangyao Zhai, Yujie Zhou

**Affiliations:** ^1^Department of Cardiology, Beijing Anzhen Hospital, Beijing Institute of Heart Lung and Blood Vessel Disease, Capital Medical University, 100029 Beijing, China

**Keywords:** serum uric acid, hyperuricemia, postmenopausal women, coronary artery disease

## Abstract

**Background:**

Serum uric acid (SUA) levels has been considered a possible risk factor for coronary artery disease (CAD) for many years. Since SUA levels are greatly affected by medications, diet, and metabolism, the association between SUA and CAD has been controversial for centuries. While, the state of hyperuricemia (HUA) has been proven to have a negative impact on CAD in previous studies, there are still few clinical and epidemiological studies of HUA in CAD. In particular, evidence of this association is limited in postmenopausal women. This study explored the influence of SUA levels and HUA on CAD in this demographic group.

**Methods:**

In total, 5435 postmenopausal women were allocated to either a non-CAD group (n = 2021) or a CAD group (n = 3414). Regression analyses, including generalized linear models (GLM), correlation analysis, comparison between stratified groups, and analysis by use of diuretics were carried out on data obtained in this study.

**Results:**

SUA and HUA were found to associate significantly with CAD by univariate logistic regression analysis. In addition, GLM showed nonlinear response of CAD probability with increasing level of SUA. In multivariate analysis, we found that SUA and HUA were independently related to CAD. Correlation analysis showed that SUA and HUA both correlated positively with CAD (*p *< 0.001). By comparing the stratified age groups, we found that the differences among the age groups were significant (*p *< 0.05).

**Conclusions:**

SUA and HUA were shown to be independently associated with CAD among postmenopausal women.

## 1. Introduction

Although the exact pathogenic role of serum uric acid (SUA) remains to be determined, many prospective cohort studies have found that SUA levels positively correlate with cardiovascular risk [1]. Over the past few decades, average SUA levels and the prevalence of hyperuricemia (HUA) in the general population appear to have greatly increased. These trends are likely related to several contributing factors, such as metabolic syndrome, obesity, lifestyle, and drug use [2]. The increase in nutrient intake in modern society may explain the increasing level of SUA [3]. Although there has been a gradual increase in evidence regarding effective therapies to lower SUA, whether SUA levels should be lowered to reduce cardiovascular disease (CVD) risks remains controversial. Some researchers have reported that hormone replacement therapy (HRT) may be associated with lower SUA levels in postmenopausal women, suggesting that estrogen may affect these levels. However, there are no indications for the treatment of asymptomatic HUA [4, 5]. In women and people with higher heart disease risks particularly, there are close links between SUA and cardiovascular events [6]. In premenopausal women, SUA levels may be predictors for major cardiovascular incidences, while in postmenopausal women, SUA correlates with higher risk of death and arterial embolism, independent of other cardiovascular risk factors and time of menopause [7, 8]. Furthermore, SUA levels are also associated with many cardiovascular risk factors, such as metabolic syndrome, hypertension, and dyslipidemia, and there is evidence SUA might also predispose postmenopausal women to endothelial dysfunction [9, 10]. Although the causal relationship between HUA and cardiovascular disease remains controversial, there is growing interest in SUA due to the worldwide prevalence of HUA [11]. In recent years, the prevalence of HUA has been rising, and it has shown a significant upward trend in postmenopausal women. Better management of the related factors might help prevent further increases in the burden of HUA for postmenopausal women [12]. Therefore, it is necessary to explore the role of SUA and HUA as disease risk factors, particularly in postmenopausal women.

## 2. Methods

### 2.1 Study Patients

We conducted a single-center retrospective study. The study population was selected from all postmenopausal women over 50 years old who underwent coronary angiography in our hospital from October 2014 to October 2015. All patients underwent coronary angiography and were grouped into CAD and non-CAD group according to the results of coronary angiography (two experienced clinicians read the angiography results). Any vessel stenosis greater than 50% was classified as CAD. The non-CAD group included 2021 patients, and the CAD group included 3414 patients. Patients with acute or chronic renal insufficiency or an abnormal level of creatinine (Cr); patients without measurements of SUA; and patients without angiographic images (**Supplementary Fig. 1**) were excluded. We defined SUA levels exceeding 360 μmol/L in measurements on different days as HUA [13, 14, 15].

### 2.2 Data Collection

Data collection included patient clinical and demographic characteristics, including body mass index (BMI); age; blood pressure, including diastolic blood pressure (DBP) and systolic blood pressure (SBP); medical history, including hypertension (HT), diabetes mellitus (DM), hyperlipidemia, HUA, family history of CVD; smoking history; drinking history, and medication history.

### 2.3 Laboratory Analyses

All blood samples were collected after the patients had fasted overnight. Measurements of SUA, triglycerides (TG), high density lipoprotein cholesterol (HDL-C), low density lipoprotein cholesterol (LDL-C), and total cholesterol (TC) were assessed via routine clinical laboratory methods.

### 2.4 Statistical Analysis

Continuous variables are displayed as mean ± standard deviation and analyzed with unpaired t-tests for normal distribution data. Categorical variables are shown as frequencies (percentages) and analyzed with chi-square tests. Pearson’s correlation was used to analyze the relationship between two continuous normally distributed variables, and Spearman’s analysis was used to analyze correlation between non-normally distribution variables. Univariate and multivariate logistic analyses were employed to calculate the adjusted odd ratio (OR) values and confidence intervals (CI) for potential risk factors. The fitting curve drawn by R package “ggplot2” was used to show logistic regression between SUA and CAD status under a generalized linear model (GLM). We established four multivariate regression models to identify the differences between SUA and HUA. Model 1 included the baseline information, model 2 included model 1 and the medical history, model 3 included model 2 and the laboratory data, model 4 included model 3 and medical therapy. Continuous variables among the age groups were analyzed by Kruskal-Wallis assessments. Two-tailed *p* values < 0.05 were deemed statistically significant. We used SPSS (version 22.0, SPSS Inc., Chicago, Illinois, USA) and R (4.0.0, https://www.r-project.org/) software for Windows for all statistical analyses.

## 3. Results

### 3.1 Features of Baseline Characteristics

At baseline, there was a significant difference between the CAD group and the non-CAD group with regards to multiple factors, including age, BMI, SBP, DBP, smoking, DM, HT, hyperlipidemia, and HUA (*p *< 0.05). The SUA level in the CAD group was significantly higher than in non-CAD group (323.58 ± 93.47 vs 293.01 ± 74.69, *p *< 0.001). At the same time, the number of individuals with HUA in the CAD group surpassed that in the non-CAD group (953 (27.91) vs 347 (16.36) *p *< 0.001) (Table 1).

**Table 1.** **Baseline characteristics of the non-CAD and CAD group**.

		Total	non-CAD	CAD	*p* value
		(n = 5435)	(n = 2021)	(n = 3414)	
Age, years		64.33 ± 7.47	63.58 ± 7.47	64.77 ± 7.44	<0.001
BMI, kg/$m^{2}$		25.81 ± 4.01	25.45 ± 3.73	26.02 ± 4.15	<0.001
SBP, mmHg		129.87 ± 15.40	129.12 ± 14.30	130.32 ± 16.00	0.005
DBP, mmHg		73.97 ± 9.44	73.58 ± 8.93	74.20 ± 9.72	0.020
Smoking, n (%)		364 (66.97)	109 (53.93)	255 (74.69)	0.003
Alcohol use, n (%)		61 (11.22)	23 (11.38)	38 (11.13)	0.933
Medical history, n (%)					
	DM, n (%)	1740 (32.01)	536 (26.52)	1204 (35.27)	<0.001
	HT, n (%)	3690 (67.89)	1332 (65.91)	2358 (69.07)	0.016
	Hyperlipidemia, n (%)	2155 (39.65)	858 (42.45)	1297 (37.99)	0.001
	HUA, n (%)	1300 (23.49)	347 (16.36)	953 (27.91)	<0.001
	Family history of CVD, n (%)	112 (2.06)	45 (2.23)	67 (1.96)	0.508
Laboratory results					
	TG, mmol/L	1.72 ± 1.26	1.65 ± 1.16	1.76 ± 1.31	0.002
	TC, mmol/L	4.39 ± 1.07	4.42 ± 1.03	4.38 ± 1.10	0.111
	LDL-C, mmol/L	2.57 ± 0.89	2.58 ± 0.87	2.56 ± 0.91	0.450
	HDL-C, mmol/L	1.11 ± 0.26	1.14 ± 0.28	1.08 ± 0.24	<0.001
	SUA, μmol/L	312.21 ± 88.20	293.01 ± 74.69	323.58 ± 93.47	<0.001
	Cr, μmol/L	31.35 ± 8.36	29.04 ± 7.98	32.97 ± 8.49	<0.001
Medical treatment, n (%)					
	Aspirin	4747 (87.34)	1720 (85.11)	3027 (88.66)	<0.001
	Clopidogrel	3064 (56.37)	640 (31.67)	2424 (71.00)	<0.001
	Statins	3826 (70.40)	1379 (68.24)	2447 (71.68)	0.007
	β-blocker	3532 (64.99)	1172 (57.99)	2360 (69.13)	<0.001
	ARB	1057 (19.45)	329 (16.28)	728 (21.32)	<0.001
	ACEI	821 (15.11)	268 (13.26)	553 (16.20)	0.003
	Furosemide	119 (2.2)	15 (0.7)	104 (3.0)	<0.001
	Torsemide	230 (4.2)	26 (1.3)	204 (6.0)	<0.001
	Hydrochlorothiazide	250 (4.6)	56 (2.8)	194 (5.7)	<0.001
	Indapamide	39 (0.7)	19 (0.9)	20 (0.6)	0.184
	Spironolactone	246 (4.5)	43 (2.1)	203 (5.9)	<0.001

CAD, coronary artery disease; BMI, body mass index; SBP, systolic blood pressure; DBP, diastolic blood pressure; DM, diabetes mellitus; HT, hypertension; HUA, hyperuricemia; CVD, cardiovascular disease; TG, triglyceride; TC, total cholesterol; LDL-C, low-density lipoprotein cholesterol; HDL-C, high-density lipoprotein cholesterol; SUA, serum uric acid; Cr, creatinine; ARB, angiotensin receptor blocker; ACEI, angiotensin converting enzyme inhibitor.

### 3.2 Univariate Logistic Analysis

**Fig. 1.**
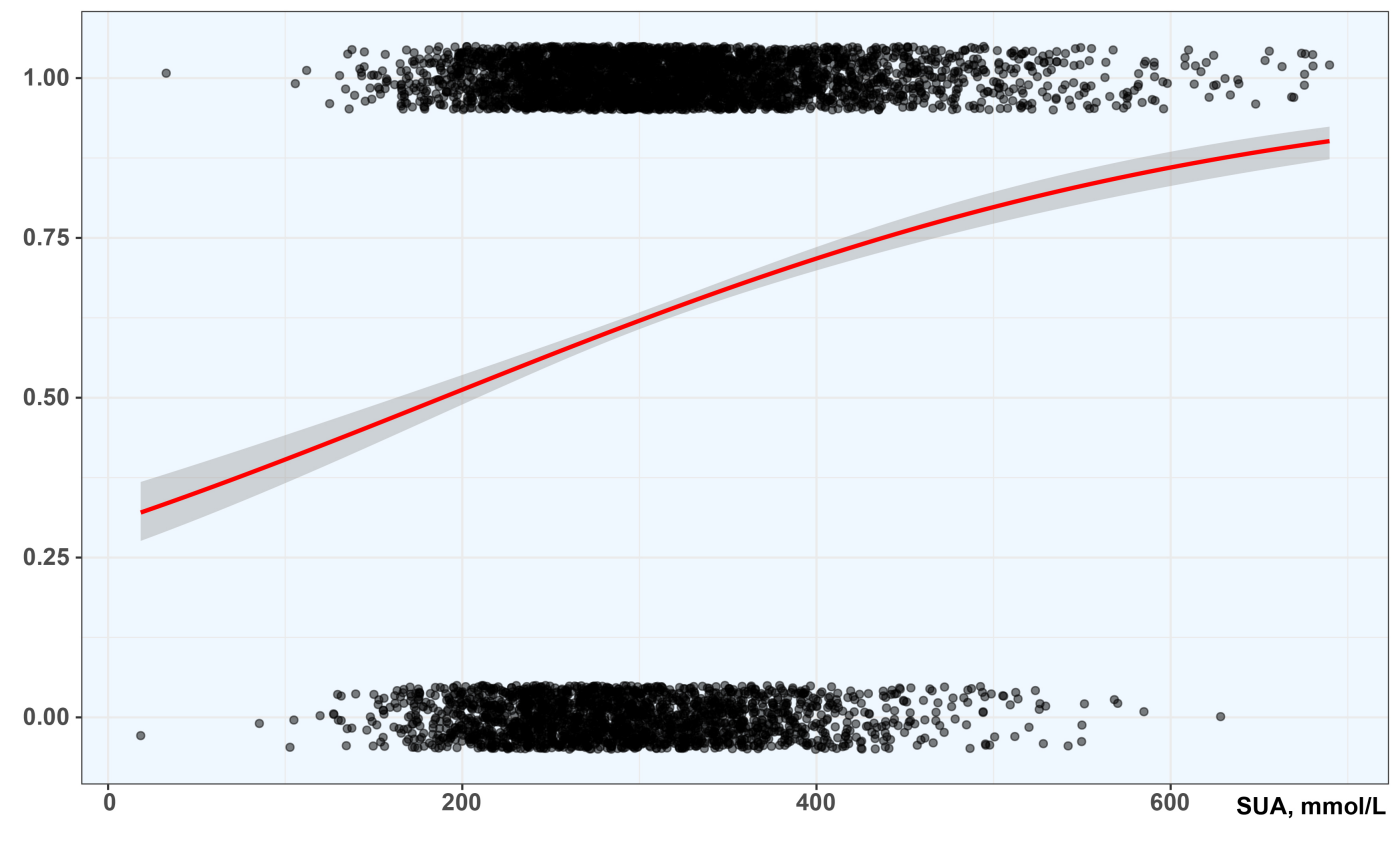
**Fitting curve obtained by logistic regression under GLM**. SUA, serum uric acid. The Y-axis shows the nonlinear response of CAD probability. The X-axis shows the SUA level.

SUA and HUA were both found to be significantly related with CAD in univariate logistic regression (SUA: OR 1.369, 95% CI 1.164–1.609, *p *< 0.001; HUA: OR 1.868, 95% CI 1.628–2.144, <0.001). Traditional risk factors for CAD, including age, BMI, DM, HT, and smoking, had significantly positive effects on CAD (*p *< 0.05). Laboratory test results, including TG and HDL-C also had significantly positive effect on CAD, while TC and LDL-C did not. We used a generalized linear model to examine the association between SUA and CAD. The gray points in the figure show the corresponding relationship between the level of SUA and CAD status of patients in the original data set (non-CAD = 0, CAD = 1). The red curve is the probability fitting curve obtained by logistic regression, which shows the nonlinear response of CAD probability (Y-axis) with the increase of SUA level (X-axis). With the increase of SUA level, more patients have CAD (Fig. 1).

### 3.3 Multivariate Logistic Analysis

**Fig. 2.**
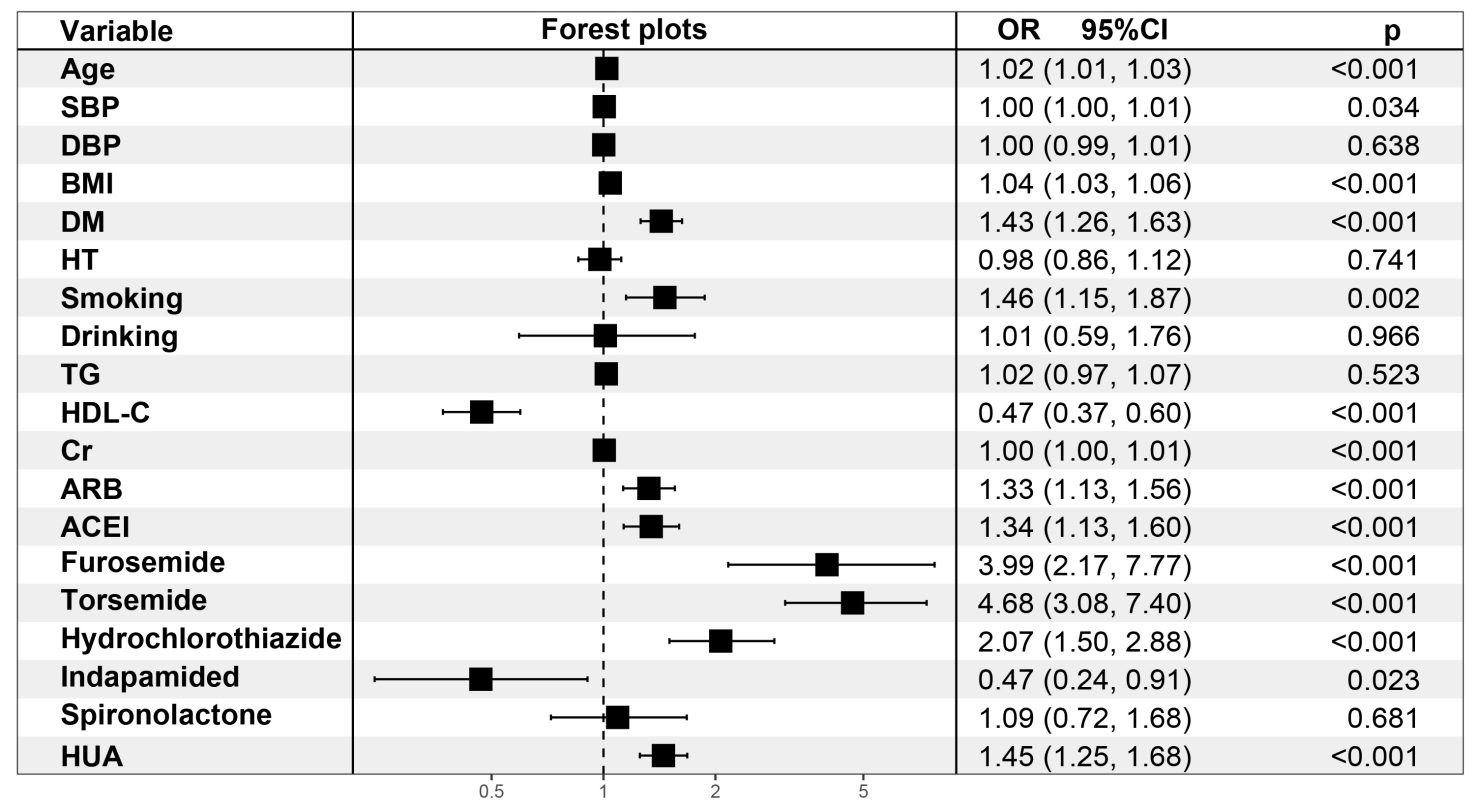
**Multivariate logistic regression analyses for HUA (Model 4)**. SBP, systolic blood pressure; DBP, diastolic blood pressure; BMI, body mass index; DM, diabetes mellitus; HT, hypertension; TG, triglycerides; HDL-C, high density lipoprotein cholesterol; Cr, creatinine; ARB, angiotensin receptor blocker, ACEI, angiotensin converting enzyme inhibitor; OR, odds ratio; CI, confidence interval; HUA hyperuricemia.

In all four multivariate regression models, SUA and HUA were found to be independently associated with CAD (*p *< 0.001). Univariate logistic regression analyses revealed the ORs of SUA were close to 1 while the ORs of HUA were higher in each of the four models (Table 2). Univariate regression of Model 4 with specific data are shown in Fig. 2 and **Supplementary Fig. 2**.

**Table 2.** **Univariate and multivariate logistic regression analyses for presence of CAD**.

	SUA			HUA		
	OR	95% CI	*p* value	OR	95% CI	*p* value
Univariate	1.369	1.164–1.609	<0.001	1.868	1.628–2.144	<0.001
Model 1	1.004	1.004–1.005	<0.001	1.844	1.605–2.119	<0.001
Model 2	1.004	1.004–1.005	<0.001	1.837	1.598–2.113	<0.001
Model 3	1.004	1.003–1.005	<0.001	1.669	1.447–1.925	<0.001
Model 4	1.003	1.002–1.004	<0.001	1.450	1.252–1.679	<0.001

OR, adjusted odds ratios; CI, confidence interval; SUA, serum uric acid; HUA, hyperuricemia. Model 1: adjusted age, BMI, SBP and DBP; Model 2: adjusted Model 1, smoking, drinking, DM and HT; Model 3: adjusted Model 2, TG, HDL-C and Cr; Model 4 adjusted Model 3, using of ARB, ACEI, furosemide, torsemide, hydrochlorothiazide, indapamide and spironolactone.

### 3.4 Correlation Analysis

Analysis of the correlation between CAD risk factors and SUA and HUA found that age, smoking, HT, and use of diuretics including all five types of diuretics were significantly positively correlated with SUA and HUA (*p *< 0.05). SUA and HUA were also significantly positively correlated with CAD (*p *< 0.001) (Tables 3 and 4).

**Table 3.** **Correlation analysis for SUA and HUA**.

	SUA		HUA	
	R	*p* value	R	*p* value
Age	0.068	<0.001	0.065	<0.001
BMI	–0.013	0.325	–0.014	0.296
SBP	–0.009	0.526	–0.012	0.380
DBP	0.021	0.126	0.023	0.091
Smoking	0.039	0.004	0.033	0.016
Drinking	0.002	0.886	–0.007	0.631
DM	–0.006	0.651	0.015	0.281
HT	0.094	<0.001	0.076	<0.001
Hyperlipidemia	0.990	<0.001	0.013	0.345
family history of CVD	0.016	0.238	–0.005	0.689
Furosemide	0.094	<0.001	0.096	<0.001
Torsemide	0.145	<0.001	0.144	<0.001
Hydrochlorothiazide	0.115	<0.001	0.110	<0.001
Indapamide	0.036	0.007	0.024	0.078
Spironolactone	0.150	<0.001	0.158	<0.001

BMI, body mass index; SBP, systolic blood pressure; DBP, diastolic blood pressure; DM, diabetes mellitus; HT, hypertension; HUA, hyperuricemia; CVD, cardiovascular disease; SUA, serum uric acid.

**Table 4.** **Correlation analysis of SUA and HUA for CAD**.

	CAD	
	R	*p* value
SUA	0.160	<0.001
HUA	0.122	<0.001

CAD, coronary artery disease; HUA, hyperuricemia; SUA, serum uric acid.

### 3.5 Stratification by Age

**Fig. 3.**
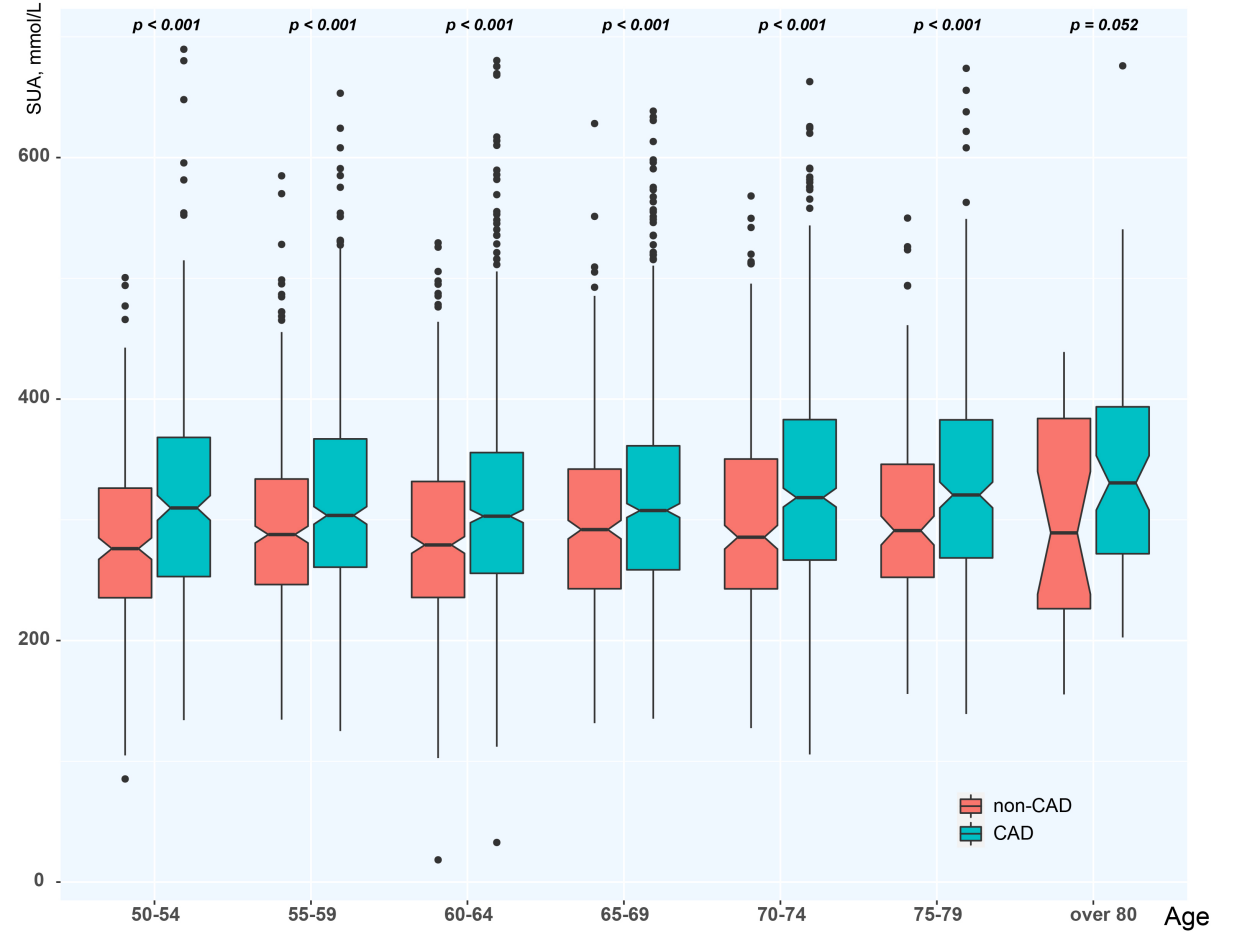
**Age stratification of SUA**. CAD, coronary artery disease; SUA, serum uric acid.

After age stratification by 5-year intervals, we found that as age increased, overall SUA levels gradually increased from the initial 303.85 ± 92.66 to 330.11 ± 93.43. SUA levels of CAD and non-CAD patients differed among age groups <80 years (*p *< 0.001), and tended to differ in the oldest group (≥80) as well (*p* = 0.052) (Fig. 3). In the comparison among groups, there were significant differences between the combined 50–54 and 55–59 age groups and the combined 70–74 and 75–79 age groups (*p *< 0.05) (Table 5). It was apparent that age had a greater impact on SUA levels in postmenopausal women with CAD than in those without CAD.

**Table 5.** **Kruskal-Wallis test of SUA between seven age groups**.

	SUA
	*p* value
50–54 vs 70–74	0.001
50–54 vs 75–79	0.002
60–64 vs 70–74	<0.001
60–64 vs 75–79	0.003

CAD, coronary artery disease; SUA, serum uric acid.

### 3.6 Use of Diuretics

Among non-CAD and CAD groups, we further divided the population into non-HUA and HUA groups, and assessed the commonly used diuretics in the different population categories. The most and least common diuretics were thiazide (3.71%) and loop (2.03%) diuretics in the non-CAD control group, while in the CAD population, they were loop (8.99%) and potassium-sparing (5.95%) diuretics. In the non-CAD and CAD groups, there were no significant differences between indapamide compared to the thiazide diuretics. The remaining three categories included four significantly different diuretics (*p *< 0.001), indicating that diuretic use was significantly higher in the CAD group as compared to the non-CAD group. For specific usage ratios, refer to **Supplementary Fig. 3**.

## 4. Discussion

### 4.1 Association between SUA and CVD in Postmenopausal Women

#### 4.1.1 SUA Levels and CAD

SUA may cause coronary endothelial dysfunction through inflammation, which could be related to early coronary atherosclerosis [16]. In the last century, researchers have paid much attention to SUA and its relationship with CAD. The SUA clearance rate was found to be faster in women than in men: 12–67 mL/min in women compared to 10–38 mL/min in men [17]. The initial study found that, although the baseline level of SUA correlated with a higher risk of primary CAD events, this association was not related to other risk factors, and changes in SUA levels did not lead to a reduction in the overall CAD risk during follow-up [18]. With increasing SUA in postmenopausal women of all age groups, especially the 55–64 age group, all causes of CVD deaths and CAD deaths were related to SUA levels [19], and there was an independent and significant correlation between the risk of cardiovascular death and elevated SUA levels [20]. In our cross-sectional study, SUA levels differed significantly between CAD and non-CAD groups, and multivariate logistic analyses found that SUA could be an independent risk factor in CAD among postmenopausal women. In addition, generalized linear models also indicated that the higher the SUA was, the more patients had CAD.

However, it has been established that metabolism levels tend to change with increasing age. Some researchers have reported that the risk of CAD increased with increasing SUA, and that there was an upward trend with aging in women [21]. In addition, SUA has been identified as an important determinant of different outcomes in cardiovascular diseases [22]. Menopause may also lead to problems in SUA excretion and thus increased SUA levels [23]. In our study, we found that age was positively correlated with SUA and HUA (r = 0.068, *p *< 0.001). Therefore, we divided age into 7 groups and explored the differences in uric acid levels between the CAD and non-CAD groups of different age groups. We found that there were significant differences between the 50 to 80 age groups (*p *< 0.05). Through regression analysis, all possible influencing factors including age were included in the multi-factor logistics models. In Model 1 to Model 4, although the OR value of SUA was greater than 1 (*p *< 0.001), it was very close to 1, suggesting that considering various factors including age, SUA was independently related with CAD.

#### 4.1.2 SUA and Hypertension in Postmenopausal Women

Nearly 100 years of studies have confirmed that the association between increased SUA and increased risk of CVD was indisputable, but the causal relationship between SUA and CVD has not been proven [24]. Previous studies have found that increased SUA levels are associated with increased risk of HT, among which HUA may be caused by direct activation of insulin resistance and vascular smooth muscle cell proliferation [25]. According to different contemporary guidelines on blood pressure, elevated SUA is associated with HT in otherwise healthy people [26]. In previous studies, SUA was positively correlated with not only the risk of hypertension, but also the risk of atherosclerosis, CVDs and metabolic syndrome [27]. In addition, a recent meta-analysis showed that elevated SUA was significantly associated with increased risk of cardiovascular death and CAD in hypertensive patients [28]. However, SUA may differ in different ethnic groups and between men and women [29]. Some studies have found that postmenopausal women have higher SUA than premenopausal women, and that a high quartile SUA may be an independent risk factor for HT in postmenopausal women [30]. Our results also suggest that SUA and HUA are significantly correlated with CAD in postmenopausal women. Estimates of SUA can improve overall risk stratification for essential hypertension, but large randomized trials are still needed to assess whether lowering SUA improves cardiovascular outcomes in patients with hypertension [31].

#### 4.1.3 SUA Excretion Promoted by Estrogen

Epidemiological studies have shown that HRT could significantly reduce the risk of CVD in postmenopausal women, probably because estrogen improves the renal clearance of SUA [32, 33]. Subsequent research found that hormone levels in women during menopause were significantly different from those before menopause. The use of estrogen during HRT might change the state of renal tubular activity, thereby inhibiting SUA reabsorption or increasing its secretion, leading to a significantly increased clearance of SUA [34]. Although estrogen is known to have protective properties for the cardiovascular system, the mechanisms are still unclear and could be the result of the beneficial effects of estrogen on blood lipids and lipoproteins [35]. In contrast, other studies have found that SUA levels only predict general and cardiovascular mortality in postmenopausal women who do not use HRT, but have not found a significant correlation between SUA and mortality in those who do use HRT [36]. The use of HRT for the primary or secondary prevention of CAD has not been supported by current evidence, and the application of HRT should be individually tailored based on the symptoms and overall risk profile of each patient [37]. Our study did not include data on HRT. More HRT research is needed in postmenopausal women, and we anticipate more such studies in the future.

### 4.2 Women with HUA and CAD and Postmenopausal Status

#### 4.2.1 HUA and CAD

HUA was detected to be an independent risk factor for morbidity and mortality of CVD. Because HUA is an increasingly serious problem, relevant research had been necessary [6]. Elevated SUA was related to CVD, but it was not consistent with coronary artery calcium (CAC). Studies have shown that SUA could be used as a race-specific marker for CAC severity levels and to measure the progression of CAC in postmenopausal women [38]. A study to determine if SUA levels were related to subclinical coronary atherosclerosis found that HUA independently predicted the instance of calcified plaques, and, thus a higher cardiovascular risk [39]. Furthermore, HUA was an independent predictor of ischemic heart disease (IHD)-related mortality in women. Compared with women with SUA levels of <238 μmol/L, women with SUA levels of ≥416.5 μmol/L had a 4.8-fold higher mortality rate related to IHD [40]. In a large sample study of middle-aged women, the prevalence of HUA increased significantly in the postmenopausal period [41]. At the same time, the risk of myocardial infarction in HUA patients increased, and the risk for women exceeded that for men (2 times vs 1.4 times) [16]. Due to the association of HUA with an increased likelihood of myocardial infarction, SUA levels should be included in assessment of CAD susceptibility [42]. In our study, HUA accounted for 23.49% of the total population, second only to DM. Additionally, we found that HUA in the CAD group was as high as 27.91%, significantly higher than HUA of the non-CAD controls. In the multivariate regression analysis, in contrast to SUA, after taking into account the same factors as SUA, OR values of HUA are not close to 1, ranging from 1.47 to 1.84 (*p *< 0.001). Therefore, HUA was found to be independently associated with CAD.

Similarly, HRT has also been shown to have protective effects in HUA patients. Menopause was found to independently correlate with HUA, while the use of hormones by postmenopausal women correlates with lower SUA levels [43]. This was probably because estrogen improves the excretion of SUA, and HRT reduces SUA in postmenopausal women with HUA. Thus, lowering SUA in HUA patients might be one of the cardiovascular protective mechanisms by which HRT reduces the risk of CAD [44]. Therefore, the effectiveness and practicability of HRT should also be further studied in postmenopausal women.

#### 4.2.2 HUA and Diuretics

HUA was closely related to the use of diuretics. Diuretics may cause HUA, changes in blood lipids, and glucose intolerance and may increase the instance of gout by causing renal retention of urate [45]. The incidence of clinical HUA was not uniform; during oral diuretic treatment, the reported incidence of HUA varied widely, ranging from 1 to 75% [46]. In terms of the physiological mechanism of diuretics, HUA due to diuretics occurred during the depletion of extracellular fluid, which might reduce urinary secretion or accelerate SUA reabsorption after secretion [47]. Thiazine diuretics were effective drugs that cause urate retention. When using thiazide diuretics at a dose of 25 mg/d or higher, the risk of anti-gout treatment increase, and long-term conventional potassium supplementation could not prevent or reduce the abnormal glucose metabolism of hypertensive patients caused by thiazide diuretics. On the contrary, it might exacerbate SUA metabolic abnormalities [48, 49, 50]. In our study, the percentage of patients in the CAD group who used diuretics was 8.99%, of which thiazide diuretics accounted for 14.3% significantly different from the non-CAD group. In the CAD combined with HUA population, thiazide diuretics accounted for 13.8% of diuretic use. Therefore, we could not ignore the use of diuretics or other special drugs that could influence the levels of SUA in postmenopausal women.

## 5. Limitations

(a) According to previous studies, our definition of HUA might be different from clinical practice. (b) This was an observational study, and the lack of patient follow-up led to the inability to accurately determine the effects of SUA levels and HUA on the prognosis of postmenopausal women with CAD. (c) It is possible that our study did not include all factors that could influence SUA levels in postmenopausal women with CAD. (d) Therapy of anti- hyperuricemia was very important in those patients who had higher levels of SUA. Because of we lack the data on antihyperuricemic agents, we could not explore the effect of antihyperuricemic therapy in our study. (e) Patients with hormone replacement therapy could not be excluded, a major limitation in our study. (f) SUA levels might be greatly affected by age while HUA could be influenced by diuretic use, so the effects of both age and diuretic use on SUA levels and HUA should not be ignored. (g) Due to the limited electronic data, we were unable to get information on the clinical presentation at the time of coronary angiography, which may introduce some bias.

## 6. Conclusions

SUA might be affected by age and diuretics use, and the effects of both factors on SUA and HUA could not be ignored. SUA and HUA were shown to be independently associated with CAD among postmenopausal women in our study sample.

## Data Availability

The datasets used and/or analyzed during the current study will be available from the corresponding author on reasonable requests.

## Abbreviations

SUA, Serum uric acid; CAD, coronary artery disease; HUA, hyperuricemia; CVD, cardiovascular disease; HRT, hormone replacement therapy; Cr, creatinine; HT, hypertension; DM, diabetes mellitus; BMI, body mass index; DBP, diastolic blood pressure; SBP, systolic blood pressure; TG, triglycerides; HDL-C, high density lipoprotein cholesterol; LDL-C, low density lipoprotein cholesterol; TC, total cholesterol; OR, odds ratio; CI, confidence interval; GLM, generalized linear model; IHD, ischemic heart disease.

## Supplementary Material

Supplementary material associated with this article can be found, in the online version, at https://doi.org/10.31083/j.rcm2307222.
