# Supplementary material for: Serum Uric Acid and Hyperuricemia Associate with Coronary Artery Disease among Postmenopausal Women
Source: Rev Cardiovasc Med. 2022 Jun 24;23(7):222. doi: 10.31083/j.rcm2307222 (PMC11266768; doi:10.31083/j.rcm2307222)
Supplement: Supplementary file 1 [file 2153-8174-23-7-222-s1.docx]

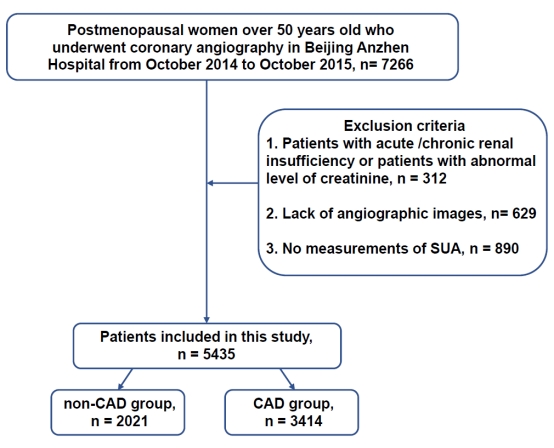


**Supplementary Fig. 1. Flow chart.**


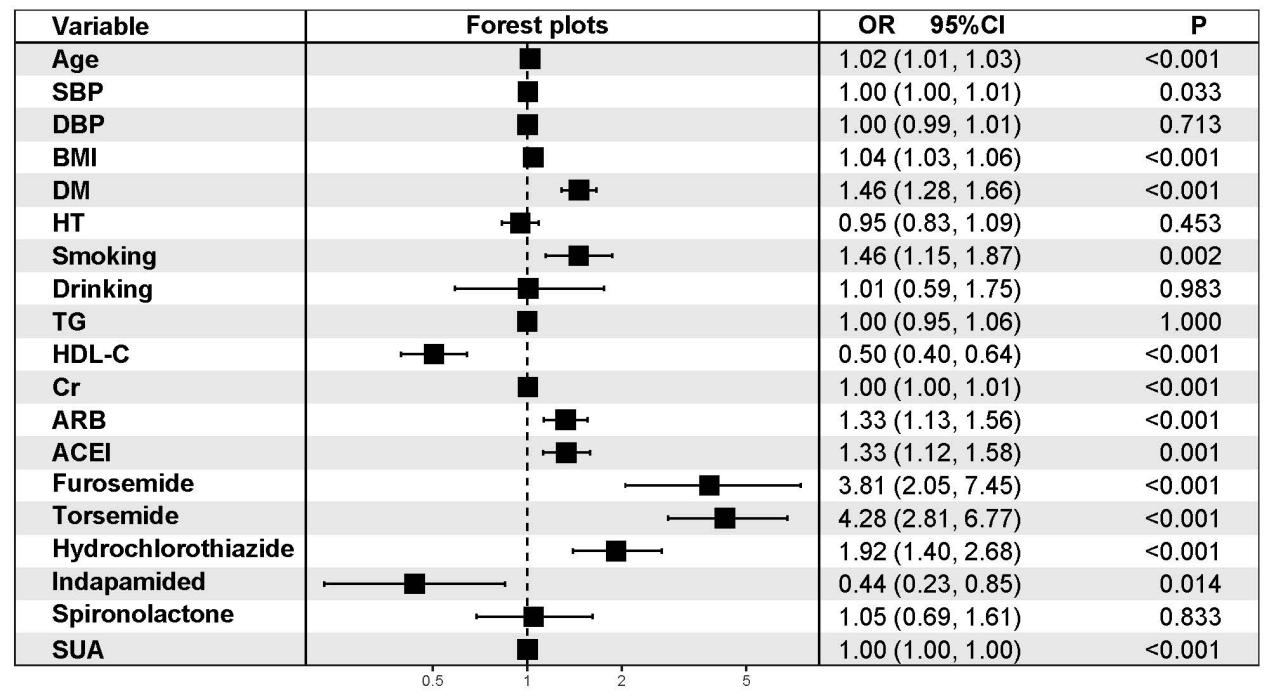


**Supplementary Fig. 2. Multivariate logistic regression analyses for SUA (Model 4)**. SBP, systolic blood pressure; DBP, diastolic blood pressure; BMI, body mass index; DM, diabetes mellitus; HT, hypertension; TG, triglycerides; HDL-C, high density lipoprotein cholesterol; Cr, creatinine; ARB angiotensin receptor blocker, ACEI angiotensin converting enzyme inhibitor; OR, odds ratio; CI, confidence interval; SUA, Serum uric acid.


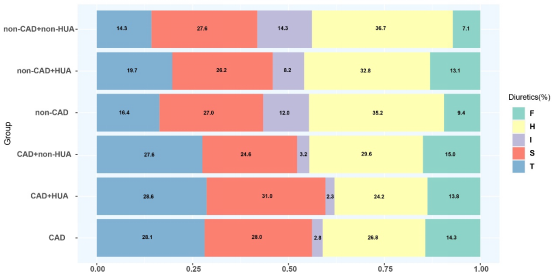


**Supplementary Fig. 3. The using ratio of diuretics in the different population.** CAD, coronary artery disease; HUA, hyperuricemia; SUA, serum uric acid; F, furosemide; H, hydrochlorothiazide; I, indapamide; S, spironolactone; T, torsemide.
